# Immunotoxicity Studies on the Insecticide 2-((1-(4-Phenoxyphenoxy)propan-2-yl)oxy)pyridine (MPEP) in Hsd:Harlan Sprague Dawley SD^®^ Rats

**DOI:** 10.3390/toxics13070600

**Published:** 2025-07-17

**Authors:** Victor J. Johnson, Stefanie C. M. Burleson, Michael I. Luster, Gary R. Burleson, Barry McIntyre, Veronica G. Robinson, Reshan A. Fernando, James Blake, Donna Browning, Stephen Cooper, Shawn Harris, Dori R. Germolec

**Affiliations:** 1 Burleson Research Technologies, Inc., Morrisville, NC 27560, USA; sburleson@brt-labs.com (S.C.M.B.); mluster@brt-labs.com (M.I.L.); gburleson@brt-labs.com (G.R.B.); 2 Division of Translational Toxicology, National Institute of Environmental Health Sciences, NIH, Research Triangle Park, NC 27560, USA; barrysmcintyre@gmail.com (B.M.); godfrey@niehs.nih.gov (V.G.R.); germolec@niehs.nih.gov (D.R.G.); 3 RTI International, Research Triangle Park, NC 27713, USA; resh@rti.org (R.A.F.); jcblake@rti.org (J.B.); dbrowning@rti.org (D.B.); sdc275@gmail.com (S.C.); 4 DLH, LLC, Bethesda, MD 20817, USA; shawn.harris@dlhcorp.com

**Keywords:** immunosuppression, immunotoxicology, MPEP, 2-((1-(4-Phenoxyphenoxy)propan-2-yl)oxy)pyridine, pyriproxyfen, pyridine, insecticide toxicity

## Abstract

The broad-spectrum insect growth regulator (IGR) and insecticide 2-((1-(4-Phenoxyphenoxy)propan-2-yl)oxy)pyridine (MPEP; also known as pyriproxyfen) is increasingly being used to address public health programs for vector control, initiated by the spread of Zika virus in 2015–2016. While considered relatively safe for humans under normal conditions, limited toxicology data are available. Current studies were undertaken to address the data gap regarding potential immunotoxicity of MPEP, with particular emphasis on host resistance to viral infection. Hsd:Harlan Sprague Dawley SD^®^ rats were treated for 28 days by oral gavage with doses of 0, 62.5, 125, 250 or 500 mg/kg/day of MPEP in corn oil. There was a dose-dependent increase in liver weights which is consistent with the liver playing a dominant role in MPEP metabolism. However, no histological correlates were observed. Following treatment, rats were subjected to a battery of immune tests as well as an established rat model of influenza virus infection to provide a comprehensive assessment of immune function and host resistance. While several of the immune tests showed minor exposure-related changes, evidenced by negative dose–response trends, most did not show significant differences in any of the MPEP treatment groups relative to vehicle control. Most notable was a negative trend in pulmonary mononuclear cell phagocytosis with increases in dose of MPEP. There was also a positive trend in early humoral immune response (5 days after immunization) to keyhole limpet hemocyanin (KLH) as evidenced by increased serum anti-KLH IgM antibodies which was followed later (14 days following immunization) by decreasing trends in anti-KLH IgM and IgG antibody levels. However, MPEP treatment had no effect on the ability of rats to clear the influenza virus nor the T-dependent IgM and IgG antibody response to the virus. The lack of effects of MPEP on host resistance to influenza suggests the immune effects were minimal and unlikely to present a hazard with respect to susceptibility to respiratory viral infection.

## 1. Introduction

2-((1-(4-Phenoxyphenoxy)propan-2-yl)oxy)pyridine (MPEP; also known as pyriproxyfen) is a broad-spectrum insect growth regulator (IGR) with insecticidal activity against certain insect pests such as houseflies, mosquitoes, and cockroaches. MPEP affects the endocrine system of insects by mimicking the juvenile hormone, thereby hindering molting and subsequently inhibiting reproduction. IGRs are unique in that they are specific for insects and have very low mammalian toxicity. Because of its high level of effectiveness against mosquitos [1] and overall low non-target toxicity [2], MPEP is considered suitable for integrated pest management programs. More recently, MPEP has been used for mosquito control in drinking water storage tanks where viral vector control is critical to public health [3], especially in developing countries lacking municipal water supplies and treatment. The U.S. EPA’s Reduced Risk Committee has granted reduced-risk and Organophosphate Alternative status for use of the insecticide to control scale insects on a variety of crops [4]. MPEP is the only pesticide approved by the World Health Organization for treatment of potable water (<0.01 mg/L) against mosquitoes [3] and has a recommended acceptable daily intake of MPEP of 100 micrograms per kg of bodyweight per day [2]. To address public health programs for vector control, including the spread of Dengue and Zika virus, Brazil applied MPEP (2 mg/L) to drinking water sources to help control the mosquito population [5].

The European Food Safety Authority [6] and World Health Organization [3] reviews, which included short- and long-term toxicity studies in mice, rats, and dogs, indicated the liver was the most sensitive target, with increases in liver weight and changes in plasma lipid concentrations, particularly cholesterol, occurring at MPEP doses of ~120 mg/kg of bodyweight per day and above. Higher doses have been associated with other effects including anemia. Reproductive toxicity studies in rats indicate fertility and overall reproductive performance were not impaired. Although MPEP is unlikely to be a teratogen, developmental toxicity studies in rabbits reported a lowest observable adverse effect level of 100 mg/kg bodyweight per day based on an increased incidence of opening of the foramen transversarium of the 7th cervical vertebra (skeletal variation). Regarding the available neurotoxicity data [6], the no-observable-adverse-effect level for acute neurotoxicity was 300 mg/kg bodyweight based on decreased total and ambulatory motor activity in male rats. The authors of the European Food Safety Authority review [6] also concluded that based on the regulatory studies submitted for pyriproxyfen (MPEP), together with the publications from the literature where a possible link of pyriproxyfen to microcephaly was reported, there was no indication of a link between the active substance and the finding of microcephaly.

The lack of information on off-target toxicity has raised concerns about its environmental persistence and the potential for latent toxicity to nontarget organisms [2,6,7]. In particular, there is a paucity of data investigating the impact of MPEP on the immune system. To date, only a single mammalian study has been conducted addressing the potential immunotoxicity of MPEP. Immunization of mice with ovalbumin containing MPEP at doses (3–15 mM) well above the recommended acceptable daily intake resulted in enhanced production of anti-ovalbumin IgG and IgG2a following the third administration, each 3 weeks apart. This was associated with increased production of TNFα and IFNγ suggesting that inflammation may play a role in the adjuvant effects of MPEP on IgG production [8]. In addition, an in vitro study showed that MPEP increased virus-associated cell death of infected mammalian cell lines [9] suggesting the potential for altered viral host resistance. Given that MPEP is being used to control vectors of viral-induced diseases, it is important to further understand its impact on immune cells and responses to determine the risk posed to host resistance and human health.

To help address the lack of toxicological data, studies were undertaken to determine the impact of MPEP on the immune response in adult Harlan Sprague Dawley rats following 28 days of oral exposure to 0, 62.5, 125, 250 or 500 mg/kg/day of MPEP in corn oil. The same dose range was tested by the National Toxicology Program (NTP) for developmental effects in rats and no evidence of maternal toxicity or developmental toxicity was observed [10]. To provide human context to this dose range, the highest dose of 500 mg/kg/day in the rat would equate to a human equivalent dose of approximately 80 mg/kg/day based on allometric scaling [11]. This human equivalent dose is over 800 times greater than the upper limit of the acceptable daily intake established for humans [3]. Parameters that were evaluated in the current studies included measurement of antibody production, cytotoxic T-cell (CTL) activity, T-cell proliferation, natural killer (NK) cell activity, macrophage function, enumeration of splenic immune cell phenotypes and host susceptibility to influenza infection. These data were used to determine the potential of MPEP to induce immunotoxicity as part of the comprehensive hazard assessment for this compound.

## 2. Materials and Methods

These studies were conducted in compliance with the U.S. Food and Drug Administration Good Laboratory Practices for Nonclinical Laboratory Studies (Title 21 of the Code of Federal Regulations, Part 58). All experiments followed the approved study protocol and applicable Burleson Research Technologies Inc. (Morrisville, NC, USA) standard operating procedures.

### 2.1. Test Materials and Dose Formulations

The test article MPEP (Lot No JL44164; CAS RN^®^ 95737-68-1) was procured from AK Scientific Inc. (Union City, CA, USA) and stored at room temperature. Comprehensive chemical analyses using gas chromatography with flame ionization detection (GC/FID) and ultra-performance liquid chromatography with photodiode array detection (UPLC/PDA) demonstrated that the bulk MPEP was ≥98% purity. Dose formulations of MPEP were prepared by RTI International (Durham, NC, USA) in corn oil (Welch, Holme & Clark Co., Inc.; Newark, NJ, USA; Lot No. 0120-0576) and provided in daily dosing bottles at a minimum volume of 60 mL/formulation in clear glass containers with a Teflon seal. Formulations at 250 mg/mL (500 mg/kg dose formulation) were considered suspensions and were mixed to homogenize prior to administration to the rats. Dose formulations were analyzed for homogeneity and concentration prior to and after administration to the test system using GC/FID.

The positive control for the immune function studies was cyclophosphamide (CPS) and was obtained from Sigma Aldrich (CAS RN^®^ 6055-19-2, Lot MKBS0021V; St. Louis, MO, USA). Selection of CPS as the positive control for the immune function testing was based on historical use and data at Burleson Research Technologies, Inc. and NIEHS, as well as extensive use as a positive control in the published literature. Dose formulations were prepared in 0.9% normal saline (Lot No. Y002998; Baxter; Deerfield, IL, USA) vehicle by RTI International (Durham, NC, USA). The identity of CPS was confirmed by infrared and nuclear magnetic resonance spectra (proton, ^13^C and ^31^P) and the purity (>99%) was determined by high performance liquid chromatography with evaporative light scattering detection (HPLC/ELSD). The pre-dose formulation and post-dose animal room samples were analyzed for concentration using UPLC/CAD (charged aerosol detector).

The positive control for the influenza host resistance study was dexamethasone and was obtained from Sigma Aldrich (CAS RN^®^ 2392-39-4, Lot MKBS2101V). Dexamethasone is an immunosuppressive drug with a well characterized mode of action and has been used as a positive control for demonstrating performance of the influenza host resistance model at Burleson Research Technologies, Inc. for over 25 years. Dose formulations were prepared in deionized water vehicle by RTI International. The identity of dexamethasone was confirmed by infrared and nuclear magnetic resonance spectra (proton, ^13^C and ^31^P) and the purity (≥99.9%) was determined by UPLC/PDA and HPLC/PDA. The pre-dose formulation and post-dose animal room samples were analyzed for concentration using LC/PDA.

### 2.2. Animals and Study Design

The studies were conducted at Burleson Research Technologies Inc., which is an AAALAC accredited research facility with Public Health Service Assurance. Animal procedures were approved by Burleson Research Technologies Inc.’s Institutional Animal Care and Use Committee prior to conducting these studies. Adult female Hsd:Harlan Sprague Dawley^®^ rats, purchased from Envigo (Haslett, MI, USA), were used to evaluate the effects of MPEP on the immune system. There is evidence that female mammals are more susceptible to certain viral infections, including Zika virus [12]. Healthy animals were randomized to treatment groups to normalize bodyweight across groups. Randomization was performed using the randomization module within Provantis v9.3.2.1. Rats were administered MPEP by gavage using 2.0 mL/kg bodyweight volumes for 28 consecutive days at concentrations of 0, 31.25, 62.5, 125, or 250 mg/mL MPEP to provide doses of 0, 62.5, 125, 250 and 500 mg/kg/day. Technical staff were aware of the group assignments to facilitate accurate treatment. The doses tested were selected for consistency with previous NTP studies examining developmental effects in rats where no evidence of toxicity was observed [10]. The immunotoxicity assessments included a positive control group (8 rats per cohort) administered 15 mg/kg CPS via intraperitoneal (IP) injection for 4–14 days (cohort dependent; see Table 1) prior to scheduled euthanasia. For the influenza host resistance study, a positive control group was administered dexamethasone by gavage at a dose of 0.6 mg/kg/day from Days 3 to 7. The positive control group showed increased susceptibility to viral infection with significant bodyweight loss and clinical signs of illness. As a result, the dose of dexamethasone was reduced to 0.2 mg/kg for Days 8–27.

Seven (7) cohorts of adult female rats were used to conduct the immunotoxicity testing and influenza host resistance studies for MPEP (Table 1). Each cohort consisted of 12 rats per vehicle and MPEP treatment groups and 8 rats for the positive control group with the exception of cohort 7 (influenza host resistance study). Cohort 7 required 10 animals per treatment group (vehicle, MPEP, and dexamethasone) for each timepoint used to determine viral clearance (Days 7, 8, 10, 14, and 28).

Group sizes were determined based on historical data from Burleson Research Technologies Inc. and NIEHS. Animals were housed in individually ventilated caging up to 3 per cage and provided NTP-2000 feed and tap water ad libitum, and Crink-l’Nest enrichment (The Andersons; Maumee, OH, USA). The housing environment was maintained at a mean temperature of 22.3 °C (standard deviation of 1.2 °C) and a mean relative humidity of 50.9% (standard deviation of 10.1%). All animals were euthanized by CO_2_ inhalation using 100% CO_2_ introduced at 3.65 L/minute into a 7.3 L chamber to displace 50% of the atmosphere per minute until breathing ceased and no pedal reflex was observed. Severing the diaphragm was used for confirmation of death.

### 2.3. Immunizations and Infections (Cohorts 2, 3, 5, 6, 7)

Animals from cohort 2 were immunized with sheep red blood cells (SRBC) in Alsever’s solution (Colorado Serum Company; Denver, CO, USA) at 1 × 10^8^ SRBC/rat via intravenous injection on Day 25 (4 days prior to scheduled euthanasia).

Animals from cohort 3 were immunized with 300 μg/rat of KLH (GMP-grade whole subunit; Stellar Biotechnologies; Port Hueneme, CA, USA) via intraperitoneal injection (IP) on Day 15 (14 days prior to scheduled euthanasia).

Animals from cohorts 5 and 6 were infected with rat-adapted influenza virus (approximately 2 × 10^5^ plaque forming units/rat) via intranasal instillation (IN) on Day 21 (8 days prior to scheduled euthanasia) and Day 26 (2 days prior to scheduled euthanasia), respectively.

Animals from cohort 7 were infected with rat-adapted influenza virus (approximately 2 × 10^5^ plaque forming units/rat) via intranasal instillation (IN) on Day 6.

### 2.4. General Toxicology and Terminal Procedures (All Cohorts)

Cageside morbidity/mortality checks were recorded at least twice daily and detailed clinical observations were conducted weekly. Bodyweights were recorded before administration and then weekly thereafter until scheduled study termination. Bodyweights were collected weekly on the same day of the week throughout the study. Bodyweight loss was monitored as a sign of toxicity. At necropsy for cohort 1 rats, animals were randomized for euthanasia order, blood was collected for hematology, then the liver, spleen, lungs, thymus, kidneys, and adrenal glands were weighed, examined, and then fixed in 10% neutral buffered formalin, along with bone marrow (femur), gastrointestinal tract with Peyer’s patches, and mesenteric and popliteal lymph nodes. Tissues were sectioned at 4–6 µm and stained with hematoxylin and eosin for histopathological evaluation. The lymphoid organs were evaluated using an enhanced histopathology guideline [13]; non-lymphoid organs were evaluated by traditional histopathology. All evaluations were conducted in accordance with the NTP Immunotoxicity Study Pathology Specifications and the pathologist was blinded to treatment group [14]. Necropsy for cohorts 2–6 included gross examination and collection of weights for spleen and thymus as well as collection of blood for serum antibody analyses (cohorts 2 and 3 only).

Groups of rats (10 per treatment group per timepoint) from cohort 7 were euthanized on Days 7, 8, 10, 14, and 28 and blood was collected, serum isolated, and stored at ≤−20 °C until analyzed for anti-influenza IgM and IgG antibodies. The lungs were removed and homogenized at 10% *w*/*v* in Eagle’s minimum essential medium supplemented with 100 U/mL penicillin and 100 μg/mL streptomycin using a FastPrep-24™ tissue homogenizer (MP Biomedicals; Solon, OH, USA). Homogenates were centrifuged for 30 min at 1000× *g* in a centrifuge set to maintain 2–8 °C. Lung homogenate supernatants were aliquoted and stored at ≤−70 °C for viral titration, and ≤−20 °C for influenza-specific IgM and IgG evaluation, as detailed below.

### 2.5. Hematology (Cohort 1)

At their scheduled termination, animals were randomized for euthanasia order, rendered unconscious with carbon dioxide (CO_2_), and blood collected. Blood (~250 μL) was collected from the retroorbital site and placed into tubes containing K_2_EDTA. Immediately following blood collection and before recovery of consciousness, the animal was returned to the CO_2_ chamber for euthanasia. The blood was analyzed the day of collection on an Advia 120 hematology analyzer using associated V.6.3.2-MS software (Siemens Medical Solutions USA, Inc., Malvern, PA, USA). The following parameters were assessed: erythrocyte count, hematocrit, hemoglobin concentration, mean cell volume, mean cell hemoglobin, mean cell hemoglobin concentration, platelet count, reticulocyte count, and white blood cell count and differential.

### 2.6. Humoral Immunity (Cohorts 2 and 3)

Humoral mediated immunity was assessed in the rats using two model T-dependent antigens, SRBC (Cohort 2) and KLH (Cohort 3). Four days following intravenous immunization of animals from cohort 2 with SRBC (1 × 10^8^ SRBC/rat in 0.5 mL saline), the T-dependent antibody response (TDAR) was assessed by measuring the number of antibody-forming cells (AFC) in the spleen, the number of IgM antibody producing B-cells (ELISpot) and the serum titers of circulating antibody to SRBC. Animals from cohort 3 received intraperitoneal immunization with KLH (300 μg/rat in 0.5 mL saline) 14 days prior to euthanasia and serum IgM and IgG antibody titers determined 5 (IgM, in-life blood sample) and 14 (IgG, terminal blood sample) days following immunization. We have described these methods in detail previously [15].

### 2.7. Pulmonary Mononuclear Cell Phagocytosis (Cohort 4)

Bronchoalveolar lavage fluid was collected, and mononuclear cells (50,000 cells) were mixed with opsonized *Staphylococcus aureus* bioparticles (100 μg) labeled with pH sensitive pHrodo™ Red fluorochrome (Invitrogen; Eugene, OR, USA) that fluoresces red within phagosomes due to the acidic pH. Cytochalasin D (15 μM; Sigma Aldrich), an in vitro inhibitor of phagocytosis, was added to selected samples for 45 min prior to the addition of bioparticles. Cells and bioparticles were incubated for 2 h (±5 min) and then cooled to 2–8 °C to stop phagocytosis. Phagocytosis was quantified as the increase in cell-associated red fluorescence (peak emission at 587 nm) using an Accuri C6^®^ flow cytometer using CFlow Plus^®^ software v1.0.264.21. Data were expressed as the percentage of cells that engulfed bacteria and the mean fluorescent intensity of cells that engulfed bacteria (measure of number of bacteria engulfed per cell).

### 2.8. Immunophenotyping in the Spleen (Cohort 4)

Splenic immune cell populations were determined using flow cytometry and are presented as absolute cell numbers and percentages of CD45+ lymphocytes for lymphoid cells or percentages of total myeloid cells, as appropriate. Red blood cells (RBC) were removed by lysis from the spleen single cell suspensions prior to blocking of Fc receptors and antibody staining. Labeled samples were analyzed on an Accuri C6^®^ flow cytometer using CFlow Plus^®^ v 1.0.264.21 (BD Biosciences; Franklin Lakes, NJ, USA). Cell populations examined in the spleen included total lymphocytes, T-cells, CD4^+^ T-cells, CD8^+^ T-cells, B-cells, NK cells, monocytes/macrophages, eosinophils, and neutrophils. In addition, B:T-cell and CD4^+^:CD8^+^ T-cell ratios were determined. Detailed procedures including antibodies, staining, and gating strategies were previously published [15].

### 2.9. Cell Mediated Immunity (Cohort 4 and 5)

Cytotoxic T Lymphocyte (CTL) effector cells were isolated from the lungs of influenza infected rats from cohort 5 and used to assess the impact of exposure to MPEP on the cell-mediated immune response [15,16]. Briefly, rats were infected eight days prior to termination with rat-adapted influenza virus, [~2 × 10^5^ plaque-forming units (PFU)/rat], via intranasal instillation. Target cells were infected with influenza virus in vitro and then labeled with Chromium-51 (^51^Cr). Lung effector cells were separated from red blood cells and adherent cells and combined with labeled target cells in U-bottomed microtiter plates at effector-to-target ratios of 50:1. 25:1 and 12.5:1. Plates were centrifuged at 250× *g* for 5 min to facilitate cell contact and incubated at 37 °C/5% CO_2_ for 6 h. Culture supernatants were harvested and release of ^51^Cr was determined using a Cobra II Auto-Gamma counter (Packard Inc., Ramsey, MN, USA). Specific target cells lysis is a direct measure of influenza-specific CTL killing activity.

T-cell proliferation in response to ex vivo treatment with monoclonal anti-CD3 antibodies was determined as previously described [17,18]. Briefly, microtiter plates were coated overnight with anti-CD3 (100 μL/well of 1 μg/mL solution of Clone G4.18; BD Biosciences) and washed. Spleen cells from cohort 4 rats were suspended in RPMI complete medium, added to the appropriate wells, and incubated at 37 °C and 5% CO_2_ for up to 96 h. Cell proliferation was determined using the Click-iT EdU Proliferation Assay (ThermoFisher Scientific, Waltham, MA, USA). EdU incorporation into DNA during proliferation results in a fluorescence signal that is directly proportional to single cell DNA content. Data were collected using an Accuri C6^®^ flow cytometer and CFlow Plus^®^ software v1.0.264.21 (BD Biosciences).

### 2.10. Natural Killer (NK) Cell Activity (Cohort 6)

Spleen effector cells were separated from red blood cells and adherent cells and the single cell suspensions were adjusted to achieve the desired effector-to-target ratios of 50:1, 25:1, and 12.5:1 [15]. Effector cells (100 μL) were added to wells of round-bottom microtiter plates containing 100 μL of YAC-1 target cells (1 × 10^5^ cells/mL labeled with ^51^Cr at 100 μCi per 1 × 10^6^ target cells for 90 min). The plates were centrifuged at 250× *g* for 5 min to facilitate cell contact and incubated at 37 °C/5%CO_2_ for 4 h. Culture supernatants were harvested and release of ^51^Cr was determined using a Cobra II Auto-Gamma counter (Packard, Inc.).

### 2.11. Titration of Influenza Virus from Lung (Cohort 7)

Infectious viral titers were measured in lung homogenate supernatants collected on Days 7, 8, 10, 14, and 28 post-infection as previously described [19]. Madin Darby Canine Kidney (MDCK) cells were used to measure infectious virus to determine viral clearance. Dilutions of lung homogenate supernatant were added to monolayers of MDCK cells and covered with an agarose overlay. Following incubation for 36–48 h to allow plaque development, the cell monolayers were fixed with neutral buffered formalin and stained with crystal violet. Viral plaques (areas of virus-mediated lysis of MDCK cells) were counted visually to determine infectious virus titer (PFU/g of lung). Viral titers were reported as log PFU/g of lung.

### 2.12. Humoral Immune Response to Influenza Virus (Cohort 7)

Influenza virus represents a T-dependent antigen, and influenza-specific IgM antibody (samples from Days 10 and 14) and IgG antibody (samples from Days 14 and 28) levels were measured in serum and lung homogenate using a hybrid enzyme-linked immunosorbent assay (ELISA). Wells were coated overnight with influenza A/Port Chalmers/1/73 (H3N2) virus grown to high titer in embryonated chicken eggs and purified by density gradient centrifugation for samples and controls, and with anti-rat IgM or IgG for standards. Plates were washed and blocked with phosphate-buffered saline-Tween 20 (0.05%) + 5% Non-Fat Dry Milk for 1 h and then blocking solution was removed. Standards, controls, serum, and lung homogenate samples from test animals were added to the plates in 100 μL volumes to appropriate wells. After washing the plates four times to remove unbound immunoglobulin, a conjugated anti-rat IgM or IgG antibody was added for 1 h. Plates were washed again four times to remove unbound conjugated antibody followed by addition of 3,3′,5,5′-Tetramethylbenzidine chromogenic substrate for 30 min in the dark. The color reaction was stopped by adding Stop Buffer and the resulting absorbance was measured using a SpectraMax i3 microplate spectrophotometer (Molecular Devices; San Jose, CA, USA). All samples were assayed in duplicate and data analysis was performed using Softmax^®^ Pro GxP version 6.5 (Molecular Devices) software. Concentrations of anti-influenza IgM and IgG were extrapolated from the standard curve using a 4-parameter fit.

### 2.13. Data Collection and Statistical Analysis

Data were collected into Provantis v9.3.2.1 and v10.2.3 (Instem, Philadelphia, PA, USA) and calculation of endpoints was performed within this validated electronic data collection and management system. Results are presented as mean ± SEM. Jonckheere’s test was used to test for dose-related trends [20]. Bodyweight and organ weight data, which typically exhibit a normal distribution, were analyzed using a parametric multiple comparison procedure. If a significant trend was detected at *p* ≤ 0.01, Williams’ test was used [21]; if the trend was not significant Dunnett’s test was used [22]. Positive control group bodyweight and organ weight data was compared to the vehicle control group using a t-test. Data for other endpoints were analyzed using a non-parametric multiple comparison procedure. If a significant trend was observed Shirley’s test was used [23]; if the trend was not significant Dunn’s test was used [24]. Positive control group data was compared to the vehicle control group using the Kruskal–Wallis test. Data that were different from control at *p* ≤ 0.05 were considered statistically significant. Prior to analyses, extreme values were identified by the outlier test of Dixon and Massey [25]. All flagged outliers were examined by NIEHS personnel, and implausible values were eliminated from the final analyses.

## 3. Results

Summary findings relevant for evaluating immune toxicity are presented below. The positive control groups treated with CPS or dexamethasone showed marked immunosuppression consistent with historical data indicating that the assays used in these studies performed as expected. All study findings including individual animal data are available at the NTP Chemical Effects in Biological Systems (CEBS) database in CEBS Summary and Individual Animal Data Tables [https://cebs-ext.niehs.nih.gov/cebs/paper/15797/private/ZjEyMTEzZTRiZDI1YTc4NzZmZDU3NzhlYTc3Yzc5NWYK (accessed on 13 July 2025)].

### 3.1. Test Chemical and Sample Analysis

Analyses confirmed the absence of MPEP in the vehicle formulation. All MPEP dose formulations analyzed prior to shipment to Burleson Research Technologies Inc. met acceptance criteria (±10% of nominal) for concentration. All dose formulations tested met the criteria for acceptable homogeneity (acceptance criteria of <5% difference between top, middle, and bottom fractions). The post-administration concentrations for the animal room samples from all MPEP dose formulations met acceptance criteria (±10% of nominal) for concentration indicating stability under the conditions and duration of use. The pre- and post-administration vehicle control samples were negative for MPEP.

Dose formulation of CPS and dexamethasone met acceptance criteria (±10% of nominal) for concentration pre- and post-administration to the rats.

### 3.2. Clinical Observations, Bodyweights, Organ Weights, and Pathology

There were no treatment-related deaths or adverse clinical observations noted in any treatment group. Clinical observations were considered sporadic or background findings based on their low incidence, minimal severity and/or similar incidence between control and treated groups (CEBS Summary Table I05). There were no treatment related effects on bodyweights (CEBS Summary Table I04) although there were slight changes in bodyweight gains (CEBS Summary Table I04G), most of which occurred during the first several weeks of treatment. At necropsy the thymus, spleen, right adrenal, right kidney, liver, lungs, femur, mesenteric lymph nodes, and popliteal lymph nodes were removed, examined, weighed (except femur, mesenteric lymph nodes, and popliteal lymph nodes), and submitted for histopathology assessment. The only treatment related effect observed in organ weights was an increase in liver weights in all rats dosed with ≥125 mg/kg MPEP (Table 2; CEBS Summary Table PA06). An increased adrenal gland weight was noted in the 500 mg/kg dose group but was attributed to a single animal with an adrenal gland weight well out of range of the remainder of the animals on study. There were no weight or histological changes in lymphoid organ or histological changes in non-lymphoid organs identified that could be attributed to oral exposure to MPEP (CEBS Summary Tables PA02, PA03, PA06, PA08, PA10, and PA46). Spleen and thymus weights were significantly decreased in the positive control (CPS) group.

### 3.3. Hematology (Cohort 1)

A complete hematological evaluation (CEBS Summary Table M04), including white blood cell (WBC) differentials (CEBS Summary Table M03), was conducted. Erythrocyte count, hematorcrit, mean cell hemoglobin, and mean cell hemoglobin concentration (MCHC) were excluded for animal 153 from the 125 mg/kg MPEP group due to being biological and statistically significant outliers (see CEBS Summary Table M04). Erythrocyte counts and hematocrits showed negative exposure-related trends while mean cell hemoglobin and mean cell hemoglobin concentration (MCHC) showed positive exposure-related trends in MPEP treated rats. Hematocrits were significantly decreased in rats treated with 500 mg/kg MPEP. MCHC was significantly increased in rats treated with ≥250 mg/kg MPEP. This effect was minor and within normal range for rats. A positive exposure-related trend occurred in reticulocyte numbers and percentages while a negative exposure-related trend occurred in eosinophil numbers and percentages, following MPEP exposure. Effects on reticulocytes and eosinophils were only statistically significant in the 500 mg/kg MPEP group. No other hematological changes were associated with MPEP exposure. Treatment with CPS resulted in marked general leukopenia.

### 3.4. Humoral Immunity (Cohorts 2 and 3)

There were no MPEP treatment-related effects on any measure of humoral immunity following immunization to SRBCs including the AFC response (CEBS Summary Table M07), serum anti-SRBC IgM titers (CEBS Summary Table M08), or the number of anti-SRBC IgM producing splenic B-cells (CEBS Summary Table M19). There were significant treatment-related effects on the serum antibody response to KLH (Table 3; CEBS Summary Table M09). The first study showed an increasing exposure-related trend and significantly increased primary anti-KLH IgM production (62.5, 250, 500 mg/kg MPEP) 5 days following immunization. By 14 days there was a negative exposure-related trend for anti-KLH IgM which was significant in rats treated with 500 mg/kg MPEP. The same negative trend was observed for anti-IgG on Day 28 (14 days following immunization) which was significantly decreased in rats treated with 500 mg/kg MPEP. However, since similar effects were not seen with SRBC, another T-dependent antigen, the response to KLH was repeated. Although the repeat study showed increases in the primary anti-KLH IgM response on Day 19 (5 days following immunization), the increases were not significant relative to the vehicle control. There were significant negative exposure-related trends 14 days following immunization in both the anti-KLH IgM and IgG antibody response like those observed in the first study (Table 3). CPS treatment resulted in a significant reduction in the KLH antibody response in both studies.

### 3.5. Cell Mediated Immunity (Cohorts 4 and 5)

Cell mediated immune functions were assessed by determining the ability of cytotoxic T lymphocytes (CTL) isolated from the lungs of influenza infected rats to destroy influenza infected target-cells (CEBS Summary Table M12) and determination of T-cell proliferation in response to plate-bound monoclonal anti-CD3 antibody stimulation (CEBS Summary Table M11). CTL responses were unaffected by MPEP treatment. Similarly, T-cell proliferation did not show any significant changes following treatment with MPEP. CPS treatment produced a significant decrease in the CTL response and caused a reduction in all parameters for T-cell proliferation, although not significant.

### 3.6. Innate Immunity (Cohorts 4 and 6)

Natural killer (NK) cell activity, a measure of innate immunity, was evaluated by examining the ability of effector cells from the spleen to lyse YAC-1 tumor target cells (Cohort 6; CEBS Summary Table M15). There were no MPEP related effects on NK cell activity. CPS treatment resulted in a decrease in NK cell activity.

**Figure 1:**
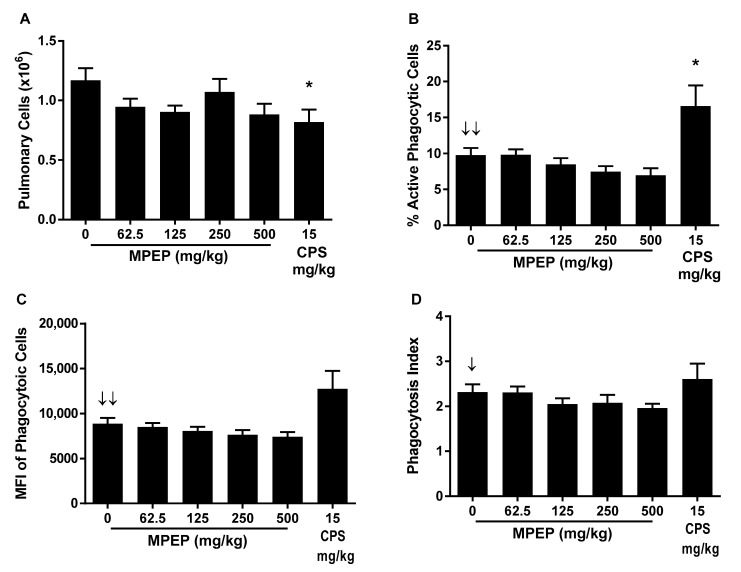
Pulmonary mononuclear cell phagocytosis of opsonized *S. aureus* rats administered 2-((1-(4-Phenoxyphenoxy)propan-2-yl)oxy)pyridine (MPEP). (**A**) Total pulmonary cells obtained per rat; (**B**) Percentage of pulmonary cells undergoing active phagocytosis; (**C**) Mean fluorescent intensity (MFI) of pulmonary mononuclear cells engulfing *S. aureus* (indicator of the number of particles engulfed per mononuclear cell); and (**D**) phagocytic index (ratio of MFI for mononuclear cells with and without bioparticles). N = 12/group for MPEP and 8/group for CPS. Significant negative exposure-related trend ↓ (*p* < 0.05) or ↓↓ (*p* < 0.01) with increasing dose of MPEP. * Significantly different from the vehicle control group (*p* < 0.05).

The impact of MPEP treatment on phagocytosis was determined by the ability of pulmonary mononuclear cells to phagocytize fluorescently labeled opsonized *Staphylococcus aureus* bioparticles (CEBS Summary Table M14). MPEP treatment did not affect the total number of pulmonary cells isolated but resulted in negative exposure-related trends in the percent of pulmonary cells that were actively phagocytizing *S. aureus* bioparticles, the mean fluorescence intensity (MFI) of the pulmonary cells (indicator of the number of particles engulfed per mononuclear cell), and the phagocytic index (ratio of MFI for mononuclear cells with and without bioparticles). However, these effects did not achieve statistical significance for any MPEP treatment group (Figure 1). CPS treatment reduced the total number of pulmonary cells but increased the percentage that were actively engulfing bioparticles without changing the phagocytic index.

### 3.7. Spleen Cell Immunophenotypes (Cohort 4)

Immune cell populations in the spleen were enumerated using flow cytometry. Immunophenotypes are presented in absolute cell numbers and as percentages of CD45+ lymphocytes for lymphoid cells or percentages of total myeloid cells, as appropriate. Cell populations examined in the spleen included total lymphocytes, T-cells, CD4^+^ T-cells, CD8^+^ T-cells, B-cells, NK cells, monocytes/macrophages, eosinophils and neutrophils. In addition, B:T-cell and CD4^+^:CD8^+^ T-cell ratios were determined (CEBS Summary Table M06). Immunophenotypic changes in the spleen resulting from MPEP exposure were unremarkable. There was no significant effect on absolute cell numbers in any immune cell type examined. Treatment related changes in relative percentages were limited to a positive but minor exposure-related trend in the relative B-cell numbers; however, none of the MPEP groups were significantly different from the vehicle control group. Rats treated with CPS demonstrated a generalized leukopenia in the spleen.

### 3.8. Influenza Virus Clearance and TDAR (Cohort 7)

**Figure 2:**
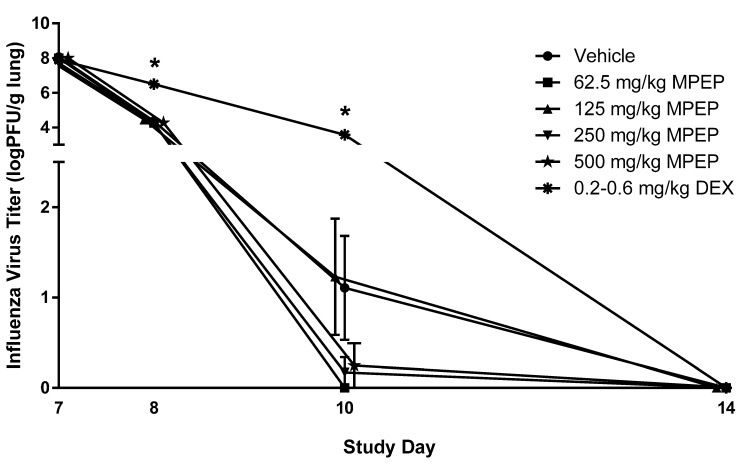
Infectious virus titer in the lungs of rats administered 2-((1-(4-Phenoxyphenoxy)propan-2-yl)oxy)pyridine (MPEP). MPEP treatment started on Day 0 and rats were infected with influenza on Day 6. Lung titers of influenza were measured on Days 7, 8, 10, 14, and 28 and all animals in all treatment groups had complete clearance by Day 14. Clearance of the virus from the lung was determined using an MDCK cell plaque assay. PFU—plaque forming unit; DEX—dexamethasone. N = 10/group/timepoint. * Significantly different from the vehicle control group (*p* < 0.05). The 500 mg/kg MPEP and 125 mg/kg MPEP data points were nudged slightly to the right and left, respectively, to improve visualization of the associated variation in response. All groups for each timepoint were euthanized and necropsied on the specified day.

Rats were infected with influenza virus on Day 6 following the initiation of MPEP treatment. To assess the impact of MPEP on host resistance, the clinical course of infection was monitored and the titer of infectious virus in the lungs was measured at Days 7, 8 10, 14, and 28. Bodyweight and lung weights were not affected by MPEP treatment (CEBS Summary Tables I04, I04G, PA06). Clearance of infectious particles from the lung was not impacted by MPEP treatment (Figure 2; CEBS Summary Table M13). It should be noted that 7/10, 10/10, 7/10, 9/10, 9/10, and 0/10 rats had fully cleared the virus by Day 10 in the vehicle, 62.5, 125, 250, 500, and dexamethasone group, respectively. The TDAR to influenza virus was also assessed as a measure of the host response to infection. Treatment with MPEP did not alter virus-specific IgM or IgG antibody titers in serum or lung homogenates (CEBS Summary Table M16). In contrast, dexamethasone treatment produced a significant delay in lung clearance of viral particles as well as lower levels of IgM and IgG antibodies in serum and lung homogenates.

## 4. Discussion

Minimal research is available in the published literature investigating the impact of MPEP on the immune system. Co-treatment of mice with ovalbumin containing pyriproxyfen (MPEP) resulted in enhanced production of anti- ovalbumin IgG and IgG2a following the third administration, each 3 weeks apart. This was associated with increased production of TNFα and IFNγ suggesting that inflammation may play a role in the adjuvant effects of MPEP on IgG production [8]. Inflammation in the brain was also observed in zebrafish exposed to MPEP for 30 days [26]. In addition, in vitro treatment of mammalian cells with MPEP increased virus-induced cell death, suggesting that host resistance may be impacted by MPEP [9]. In the present work, we undertook in-depth evaluation to determine the potential for MPEP to produce immunotoxicity and alter host resistance using an adult rat model. In general, there were minimal treatment-related effects on the immune system. Specifically, there were no treatment related effects on lymphoid organ weights or their histology, antibody responses to SRBCs, CTL activity, anti-CD3 antibody mediated T-cell proliferation, NK cell activity or spleen cell immunophenotypes. The liver was the most sensitive target in the present study, with increases in liver weight occurring at doses of ≥125 mg/kg bodyweight consistent with metabolism of MPEP in the liver [27,28]. There was some evidence that MPEP impacted the immune system based upon a decreasing trend in pulmonary cell phagocytosis as well as modulation of the humoral immune response to the T-dependent antigen KLH. There was an apparent increase in the primary IgM response to KLH in rats treated with MPEP while later antibody production showed decreasing trends for both anti-KLH IgG (60–62% reduction at 500 mg/kg MPEP) and IgM (14–34% reduction at 500 mg/kg MPEP), with IgG being more sensitive. Differential effects on antibody production with respect to antigen (SRBC vs. KLH) as well as temporal difference could imply an effect on immunoglobulin class switching (also known as. isotype switching). This involves a change in the constant-region portion of the antibody heavy chain, but not the variable region, and results in plasma cells switching predominantly from the IgM isotype to the IgG isotype. The antibody assessment to SRBCs only measured the IgM isotype while KLH response assessed both IgM and IgG. Isotype switching is a complicated process but is predominantly mediated by cytokines produced by Th1, Th2 and Treg cells including IL-4, IL-5, INFγ, TGFβ and IL-10 [29,30]. It was previously shown that MPEP increased production of TNFα and IFNγ in mice [9]. Therefore, it is possible that MPEP may modulate cytokine production/regulation and thereby influence B-cells and isotype switching, although the present study was not designed to test this hypothesis.

MPEP treatment also caused a decreased dose–response trend in pulmonary cell phagocytosis; however, the decreases did not achieve statistical significance. While the assay we employed provides a sensitive measure of phagocytic ability, it was not designed for identification of the cell type ingesting the bioparticles (e.g., macrophage, neutrophil, dendritic cells), or to determine the biocidal activity. While phagocytosis involves a cascade of events, it is interesting to note that proinflammatory cytokines, such as IL-1α, play a central role in the activation and phagocytic potential [31]. Thus, similar to isotype switching, the data suggests the potential for MPEP to disrupt cytokine regulation.

## 5. Conclusions

In conclusion, although we noted minor perturbations of innate and humoral immune responses following MPEP treatment, they were of insufficient magnitude to influence the susceptibility of rats to influenza infection. Influenza virus is a T-dependent antigen and virus clearance requires an intact and functional immune system that incorporates a cascade of immune responses including innate immunity (e.g., phagocytosis, antigen processing and presentation., NK cell activity) as well as acquired or adaptive immunity (e.g., CTL killing of infected cells, antiviral antibody production). Thus, almost all immune processes are evoked following influenza infection and potent immunotoxic compounds result in impairment of viral clearance and increased severity and/or duration of disease. As such, the lack of findings in the host resistance studies and minimal effects on other immunotoxicity endpoints suggest that there was minimal impact on immune function in rats following MPEP exposure.

## Figures and Tables

**Table 1:** Endpoints examined for each cohort.

Cohort (n)	Study Endpoints
1 (68F)	Clinical observations Bodyweights Organ weights Histopathology Hematology
2 (68F)	Clinical observations Body and organ weights Antibody response to SRBC
3 (68F)	Clinical observations Body and organ weights Antibody response to KLH—In-life, 5 days following immunization (IgM) and Terminal, 14 days following immunization (IgG)
4 (68F)	Clinical observations Body and organ weights T-cell proliferation Pulmonary mononuclear cell phagocytosis Immunophenotyping of the spleen
5 (68F)	Clinical observations Body and organ weights CTL response to influenza infection
6 (68F)	Clinical observations Body and organ weights NK response following influenza infection
7 (300F)	Clinical response to influenza infection Bodyweights Viral titers from lung at terminal necropsy on Days 7, 8, 10, 14, 28 Serum levels of anti-influenza IgM and IgG

SRBC—sheep red blood cells, KLH—keyhole limpet hemocyanin, Ig—immunoglobulin, NK—Natural Killer, CTL—cytotoxic T-lymphocyte. Cohorts 1–6 consisted of 12 rats per MPEP exposure group and 8 rats for the positive control group. Cohort 7 utilized 10 rats MPEP and dexamethasone exposure groups at each of 5 timepoints.

**Table 2:** Organ weights in rats from cohort 1 following administration of 2-((1-(4-Phenoxyphenoxy)propan-2-yl)oxy)pyridine (MPEP).

	MPEP (mg/kg)					CPS (mg/kg)
	0	62.5	125	250	500	15
Number of Rats	12	12	12	12	12	8
Bodyweight (g)	238.3 ± 2.5	239.0 ± 2.1	242.8 ± 2.2	242.4 ± 2.0	239.4 ± 2.7	233.9 ± 2.4
Thymus Weight (g)	0.327 ± 0.012	0.326 ± 0.013	0.323 ± 0.011	0.341 ± 0.015	0.327 ± 0.018	0.136 ± 0.008 *
Thymus:BW Ratio (mg/g)	1.37 ± 0.06	1.36 ± 0.05	1.33 ± 0.05	1.41 ± 0.06	1.37 ± 0.07	0.58 ± 0.03 *
Spleen Weight (g)	0.593 ± 0.020	0.546 ± 0.024	0.599 ± 0.024	0.590 ± 0.014	0.591 ± 0.030	0.387 ± 0.016 *
Spleen:BW Ratio (mg/g)	2.49 ± 0.08	2.29 ± 0.10	2.47 ± 0.11	2.43 ± 0.05	2.46 ± 0.11	1.65 ± 0.06 *
Liver Weight (g)	8.64 ± 0.18 ^↑^	8.87 ± 0.15	9.52 ± 0.20 *	9.59 ± 0.28 *	10.17 ± 0.22 *	8.33 ± 0.15
Liver:BW Ratio (mg/g)	36.24 ± 0.53 ^↑^	37.10 ± 0.54	39.23 ± 0.78 *	39.51 ± 0.96 *	42.45 ± 0.68 *	35.60 ± 0.56

Data are displayed as mean ± SEM; MPEP—2-((1-(4-Phenoxyphenoxy)propan-2-yl)oxy)pyridine; CPS—cyclophosphamide. ^↑^ Significant increasing trend with increasing dose of MPEP. * Significantly different from the vehicle control group at *p* < 0.05.

**Table 3:** KLH antibody titers in serum of rats administered 2-((1-(4-Phenoxyphenoxy)propan-2-yl)oxy)pyridine (MPEP).

	MPEP (mg/kg)					CPS (mg/kg)
	0	62.5	125	250	500	15
No. of Rats	12	12	12	12	12	8
Study 1						
IgM anti-KLH titer (Day 19)	173.59 ± 9.66 ^↑^	314.52 ± 17.55 *	283.85 ± 45.89	363.55 ± 38.93 *	362.19 ± 38.75 *	94.81 ± 15.45 *
IgM anti-KLH titer (Day 28)	105.17 ± 11.15 ^↓^	81.38 ± 7.29	109.58 ± 13.62	69.41 ± 10.94 *	69.42 ± 6.63	16.89 ± 2.09 *
IgG anti-KLH titer (Day 28)	34.571 ± 10.473 ^↓^	11.097 ± 3.569	28.895 ± 6.857	9.047 ± 2.257 *	13.755 ± 7.282 *	0.780 ± 0.000 *
Study 2						
IgM anti-KLH titer (Day 19)	82.88 ± 8.80	98.33 ± 22.30	118.28 ± 26.50	81.91 ± 10.18	106.43 ± 16.08	21.53 ± 2.95 *
IgM anti-KLH titer (Day 28)	144.94 ± 14.34 ^↓^	148.48 ± 11.02	130.71 ± 13.77	102.32 ± 14.13	124.19 ± 13.95	18.75 ± 3.06 *
IgG anti-KLH titer (Day 28)	15.646 ± 4.708 ^↓^	14.895 ± 6.180	11.612 ± 2.622	14.027 ± 6.142	5.872 ± 1.548	0.780 ± 0.000 *

Titer units are μg/mL. Data are displayed as mean ± SEM; MPEP—2-((1-(4-Phenoxyphenoxy)propan-2-yl)oxy)pyridine; CPS—cyclophosphamide, KLH—keyhole limpet hemocyanin. Significant ^↑^ increasing or ^↓^ decreasing trend with increasing dose of MPEP. * Significantly different from the vehicle control group at *p* < 0.05.

## Data Availability

All study findings including individual animal data are available at the National Toxicology Program Chemical Effects in Biological Systems (CEBS) database [https://cebs-ext.niehs.nih.gov/cebs/paper/15797/private/ZjEyMTEzZTRiZDI1YTc4NzZmZDU3NzhlYTc3Yzc5NWYK (accessed on 13 July 2025)]. These data are free to download and use.

## Abbreviations

The following abbreviations are used in this manuscript:

BW	Bodyweight
CAD	Charged aerosol detection
CEBS	Chemical Effects in Biological Systems
CO_2_	Cabon dioxide
CPS	Cyclophosphamide
CTL	Cytotoxic T-cell
ELISA	Enzyme-linked immunosorbent assay
ELSD	Evaporative light scattering detection
FID	Flame ionization detection
GC	Gas chromatography
HPLC	High-performance liquid chromatography
IFNγ	Interferon γ
IgG	Immunoglobulin G
IgM	Immunoglobulin M
IGR	Insect growth regulator
IL	Interleukin
IN	Intranasal
KLH	Keyhole limpet hemocyanin
LC	Liquid chromatography
MCHC	Mean cell hemoglobin concentration
MDCK	Madin Darby Canine Kidney
MFI	Mean fluorescence intensity
MPEP	2-((1-(4-Phenoxyphenoxy)propan-2-yl)oxy)pyridine
NK	Natural Killer
NIEHS	National Institute of Environmental Health Sciences
NTP	National Toxicology Program
PDA	Photo diode array detection
PFU	Plaque forming unit
RBC	Red blood cell
SD	Sprague Dawley
SEM	Standard error of the mean
SRBC	Sheep red blood cell
TDAR	T-dependent antibody response
TGFβ	Transforming growth factor β
TNFα	Tumor necrosis factor α
U.S. EPA	United States Environmental Protection Agency
UPLC	Ultra high-performance liquid chromatography
WBC	White blood cell
